# Plant Proteins and Processes Targeted by Parasitic Nematode Effectors

**DOI:** 10.3389/fpls.2019.00970

**Published:** 2019-07-30

**Authors:** Joffrey Mejias, Nhat My Truong, Pierre Abad, Bruno Favery, Michaël Quentin

**Affiliations:** Centre National de la Recherche Scientifique, Institut National de la Recherche Agronomique, Institut Sophia Agrobiotech, Université Côte d’Azur, Sophia Antipolis, France

**Keywords:** root-knot nematodes, cyst nematodes, galls, syncytium, effectors

## Abstract

Sedentary endoparasitic nematodes, such as root-knot nematodes (RKN; *Meloidogyne* spp.) and cyst nematodes (CN; *Heterodera* spp. and *Globodera* spp.) cause considerable damage to agricultural crops. RKN and CN spend most of their life cycle in plant roots, in which they induce the formation of multinucleate hypertrophied feeding cells, called “giant cells” and “syncytia,” respectively. The giant cells result from nuclear divisions of vascular cells without cytokinesis. They are surrounded by small dividing cells and they form a new organ within the root known as a root knot or gall. CN infection leads to the fusion of several root cells into a unique syncytium. These dramatically modified host cells act as metabolic sinks from which the nematode withdraws nutrients throughout its life, and they are thus essential for nematode development. Both RKN and CN secrete effector proteins that are synthesized in the oesophageal glands and delivered to the appropriate cell in the host plant via a syringe-like stylet, triggering the ontogenesis of the feeding structures. Within the plant cell or in the apoplast, effectors associate with specific host proteins, enabling them to hijack important processes for cell morphogenesis and physiology or immunity. Here, we review recent findings on the identification and functional characterization of plant targets of RKN and CN effectors. A better understanding of the molecular determinants of these biotrophic relationships would enable us to improve the yields of crops infected with parasitic nematodes and to expand our comprehension of root development.

## Introduction

Plant-parasitic nematodes (PPN) are microscopic worms that withdraw nutrients from the cytoplasm of cells in the aerial or below-ground parts of plants. Root-knot nematodes (RKN) and cyst nematodes (CN) are the most widely studied PPN, as these two groups are the most damaging to crop plants (Singh et al., 2013). RKN from the *Meloidogyne* genus are found throughout the world and are extremely polyphagous, infecting thousands of plant species, including both monocotyledons and eudicotyledons (Blok et al., 2008). By contrast, CN tend to specialize on a particular crop and form two common genera: *Globodera* spp. (potato CN) and *Heterodera* spp. (sugar beet, soybean, or cereal CN), each of which causes huge yield losses on its host.

**FIGURE 1:**
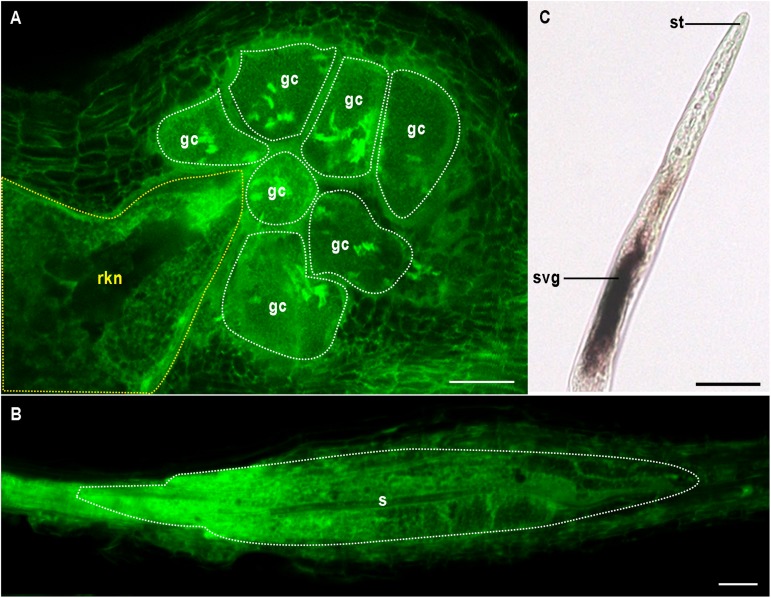
Multinucleate and hypertrophied feeding cells induced by endoparasitic plant nematodes. **(A)** Giant cells (gc and white outline) induced by the root-knot nematode *Meloidogyne incognita* (rkn and yellow outline) in *Arabidopsis thaliana*. **(B)** Syncytium (s and white outline) formed by the cyst nematode *Heterodera schachtii* in *A. thaliana*. **(A,B)** confocal images were obtained by visualizing glutaraldehyde fixative auto-fluorescence after BABB clearing as described in Cabrera et al. (2018). **(C)** *In situ* hybridisation of the pioneer *M. incognita* effector gene *Minc16401* encoding a predicted peptide of 69 amino acids (Abad et al., 2008) was performed as described in Jaouannet et al. (2018). *Minc16401* expression was localized in subventral glands (svg), suggesting the effector could be secreted *in planta* via the stylet (st). Bars = 50 μm.

Both CN and RKN are sedentary endoparasites and obligate biotrophs. Mobile preparasitic juveniles (J2) penetrate the host root, traveling toward the vascular cylinder, where they become sedentary, triggering the formation of an unusual feeding site. The RKN feeding site consists of so-called “giant cells” (Figure 1A). These cells are produced from about half a dozen vascular root cells, which undergo repeated nuclear divisions without cell division. These cells become polynucleate and may be more than 300 times larger than normal cells. Giant cells are surrounded by dividing cells, the hyperplasia and hypertrophy of which lead to the formation of a novel organ called a gall (Kyndt et al., 2013; Favery et al., 2016; Palomares-Rius et al., 2017). By contrast, CN induce the formation of a different type of feeding site called a syncytium. Syncytium formation involves partial dissolution of the root cell wall and protoplast fusion, leading to an iterative process of fusion of the first CN-infested vascular cell with its neighbors (Figure 1B; Sobczak and Golinowski, 2011; Palomares-Rius et al., 2017). Some mature syncytia are the result of fusions of more than 200 cells. Giant cells and syncytia have a number of features in common, including a fully expanded endoplasmic reticulum, a fragmented vacuole, a reorganized cytoskeleton, thickened cell walls with local ingrowths, a large mitochondrial network and endoreduplicated nuclei (Kyndt et al., 2013; Rodiuc et al., 2014). These specialized feeding cells supply the nematodes with nutrients throughout the sedentary part of their life cycle. Female RKN lay their eggs in a gelatinous matrix generally on the root surface, whereas the cyst of CN consists of a dead and hardened female containing eggs.

Root-knot nematodes and CN secrete molecules called “effectors,” to facilitate invasion of the host root, avoid plant defense responses and reprogram root cells to form specialized feeding cells. These effectors are produced principally in three oesophageal salivary glands and are then injected into plant cells via the syringe-like stylet. The activity of the oesophageal glands is developmentally regulated. The two subventral glands (SvG) secrete effectors allowing J2 penetration and migration in the root while proteins secreted during parasitism are produced by SvG and particularly by the dorsal gland (DG) (Nguyen et al., 2018). Some effectors may also be produced in other secretory organs, such as chemosensory amphids, or directly secreted through the PPN cuticle. Molecular dialog studies have focused mostly on secreted proteinaceous effectors (Hewezi and Baum, 2013; Mitchum et al., 2013; Quentin et al., 2013; Hewezi, 2015; Ali et al., 2017; Vieira and Gleason, 2019) even though other secreted molecules, such as phytohormones, have been shown to favor these interactions (Siddique and Grundler, 2018).

Various approaches have been used to characterize nematode effector repertoires. Proteomic analysis has directly identified about 500 proteins secreted by *M. incognita* preparasitic J2s or females (Bellafiore et al., 2008; Wang et al., 2012). Effector identification has greatly benefited from advances in sequencing technologies. Complete genome sequences are now available for four RKN – *M. incognita*, *M. hapla*, *M. javanica*, and *M. arenaria* (Abad et al., 2008; Opperman et al., 2008; Blanc-Mathieu et al., 2017) – and three CN: *G. pallida*, *G. rostochiensis*, and *H. glycines* (Cotton et al., 2014; Eves-van den Akker et al., 2016; Masonbrink et al., 2019). Bioinformatic methods for identifying genes encoding putative secreted proteins, which are based on the presence of a signal peptide for secretion but absence of transmembrane domains, remain the most convenient approach to identify candidate effector genes. *Cis*-regulatory sequences called “DOG boxes” were recently identified within the promoters of *G. rostochiensis* and *H. glycines* genes encoding effectors specifically expressed within the DG of the CN (Eves-van den Akker et al., 2016; Masonbrink et al., 2019). This discovery opens up new possibilities for effector prediction and implies that effector production in the DG is synchronized by master regulators, such as key transcription factors (Eves-van den Akker and Birch, 2016). Finally, transcriptomics has made it possible to compare different stages of nematode development and to identify RKN (Li et al., 2016; Petitot et al., 2016; Nguyen et al., 2018; Shukla et al., 2018) and CN (Cotton et al., 2014; Kumar et al., 2014; Yang et al., 2017; Gardner et al., 2018) genes upregulated in plants. *In situ* hybridisation (ISH) has generally been used for the initial validation of candidate effector gene expression within secretory organs (Figure 1C and Table 1). Remarkably, the secretion of a few effectors has been demonstrated *in planta*, by immunolocalisation (Table 1). Delivery to the host apoplast has been demonstrated for several effectors (Jaubert et al., 2005; Vieira et al., 2011; Iberkleid et al., 2013; Eves-van den Akker et al., 2014; Zhuo et al., 2019), but few demonstrations of translocation into the host feeding cell have been reported (Jaouannet et al., 2012; Lin et al., 2012; Chen et al., 2017; Lilley et al., 2018; Naalden et al., 2018). These studies have expanded the repertoire of putative effectors considerably, with hundreds of ISH-validated effectors now known (Truong et al., 2015; Gardner et al., 2016). However, the vast majority of these proteins are pioneer proteins with no known functional domains. As a result, the functions of only a few RKN and CN effectors have been deciphered. Cell wall-degrading effectors have been reported to help nematodes to penetrate and migrate within the root, and effectors suppressing plant defenses have been described (Quentin et al., 2013; Goverse and Smant, 2014), but only a few effectors have been shown to contribute to the *de novo* organogenesis and maintenance of feeding sites. Functional analyses of PPN effectors have clearly benefited from the identification of the host targets of these molecules, mostly through yeast two-hybrid approaches (Table 1). We review here the most recent advances in our understanding of RKN and CN effector functions, focusing on those for which the plant processes targeted have been identified.

**TABLE 1:** Nematode effectors and their identified plant targets.

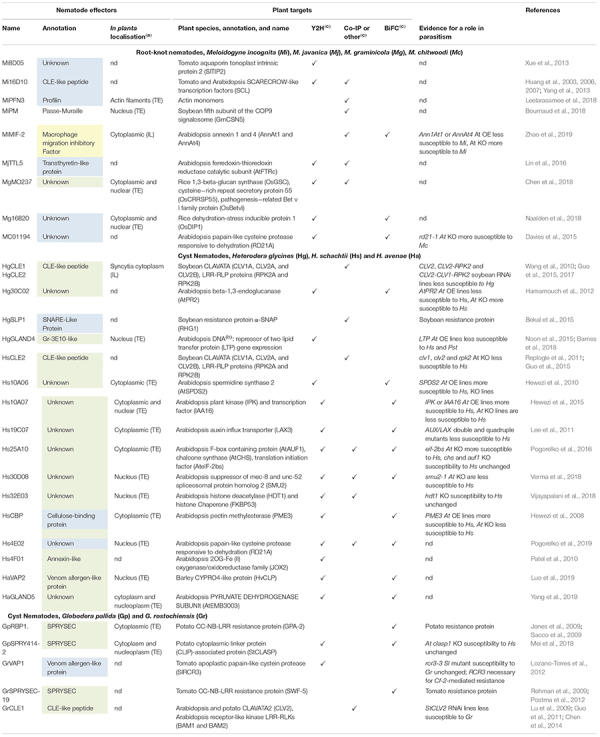

*^(a)^Effectors expressed in subventral glands (in blue), in dorsal glands (in green), and in hypodermis (in yellow); nd, not determined; IL, immunolocalisation; TE, transient expression in *Nicotiana benthamiana* leaves or Arabidopsis protoplasts. ^(b)^DNA Binding effector. ^(c)^Approaches used for target identification and validation (Y2H, yeast two hybrid; Co-IP, co-immunoprecipitation; pull-down or *in vitro* binding assay; BiFC, Bimolecular Fluorescence complementation or Luciferase Complementation). *At*, *Arabidopsis thaliana*; *Pst*, *Pseudomonas subtilis* DC3000; *Mc*, *Meloidogyne chitwoodi*; Sl, *Solanum lycopersicum*; OE lines, overexpressing lines; KO, T-DNA knockout line.*

## Parasitism Requires the Manipulation of Diverse Host Functions

Nematode effectors target the apoplast and different subcellular compartments, including the nuclei, reflecting the diversity of host cell processes manipulated to promote infection and feeding site formation (Table 1). Many of the members of the PPN effector repertoire have been shown to suppress plant immunity (Goverse and Smant, 2014; Favery et al., 2016; Ali et al., 2017). However, their precise mode of action remains largely unknown and only a few of their direct targets in plants have been identified. PPN effectors may interact with host proteins to scavenge reactive oxygen species (ROS) accumulating during the oxidative burst following the induction of pathogen-associated molecular pattern (PAMP)-triggered immunity (PTI). *M. javanica* MjTTL5 scavenges ROS by interacting with a thioredoxin reductase catalytic subunit (AtFTRc) in the plant (Lin et al., 2016). *H. schachtii* Hs10A06 has been shown to interact with a spermidine synthase (AtSPDS2), thereby enhancing spermidine production and inducing ROS-scavenging activity when the spermidine is oxidized by polyamine oxidase (Hewezi et al., 2010). Various pathogenesis-related (PR) proteins involved in the production of antimicrobial proteins by plants in response to pathogen attack have also been identified as direct targets of nematode effectors. *H. glycines* Hg30C02 targets a beta-1,3-endoglucanase (AtPR2), the inactivation of which in a mutant *Arabidopsis* line increases susceptibility to cyst nematode infection (Hamamouch et al., 2012). *M. graminicola* MgMO237 has been shown to suppress PTI by interacting with multiple host PR proteins, a 1,3-beta-glucan synthase (OsGSC), the cysteine-rich repeat secretory protein 55 (OsCRRSP55) and a pathogenesis-related Bet v I family protein (OsBetvI) (Chen et al., 2018). The GrVAP-1 effector from *G. rostochiensis* targets an apoplastic papain-like cysteine protein (PLCP) called RCR3^*Pim*^, to subvert immunity. GrVAP-1 is also recognized by a plant immune receptor called Cf-2 that can trigger effector-triggered immunity (ETI) followed by a hypersensitive response (Lozano-Torres et al., 2012).

Like other classes of plant pathogens that have to overcome host defenses, PPNs produce effectors that converge on evolutionarily conserved host targets called “hubs” (Carella et al., 2018). The *M. incognita* “Passe-Muraille” peptide effector, for example, interacts with subunit 5 of the COP9 signalosome (CSN5) (Bournaud et al., 2018), a hub targeted by bacterial, fungal and viral effectors (Mukhtar et al., 2011; Weßling et al., 2014). The function of CSN5 in RKN parasitism remains unknown, but this target protein is known to be involved in plant salicylic acid-mediated defense (Kazan and Lyons, 2014). Similarly, the *H. schachtii* Hs25A01 effector interacts with eIF-2bs, a member of the eIFs family of translation initiation factors including known host targets of fungi, bacteria and viruses, and a role for this target in parasitism was demonstrated by the observation of changes in susceptibility to nematodes in eIF-2bs knockout mutants (Pogorelko et al., 2016). A third striking example is provided by PLCPs, which constitute key hubs in plant immunity (Misas-Villamil et al., 2016). PLCPs are targeted by *M. chitwoodi* Mc01194 (Davies et al., 2015) and CN *G. rostochiensis* GrVAP-1 and *H. schachtii* Hs4E02 (Lozano-Torres et al., 2012; Pogorelko et al., 2019) effectors in diverse host plants. Mc01194 and Hs4E02 target the same *Arabidopsis* PLCP, “Responsive to Dehydration 21A” (RD21A), to promote parasitism (Davies et al., 2015; Pogorelko et al., 2019). The expression of *G. rostochiensis* VAP1, which targets RCR3^*Pim*^ in tomato, promotes susceptibility to *G. rostochiensis* and to the leaf mold *Cladosporium fulvum* (Lozano-Torres et al., 2012). It seems likely that other such molecular hubs are targeted by nematode effectors.

Other host functions targeted by RKN and CN effectors may be more related to the *de novo* formation and functioning of the specialized feeding site. The formation of feeding cells induced by RKN and CN requires a major reorganization of cytoskeletal networks (de Almeida Engler and Favery, 2011). RKN have been reported to secrete cytoskeleton components, such as actin or tubulin (Bellafiore et al., 2008), or associated proteins. A *M. incognita* profilin-like effector, MiPFN3, was recently shown to bind actin, altering its filament structure to favor parasitism (Leelarasamee et al., 2018). The *G. pallida* GpSPRY-414-2 effector, a CN-specific secreted SPRY domain-containing protein (SPRYSEC), has been shown to bind a potato microtubule-associated protein, CLASP (for cytoplasmic linker protein-associated protein) (Mei et al., 2018). CLASP proteins are involved in both cell division and cell expansion (Ambrose et al., 2007). GpSRY-414-2 therefore probably modulates the microtubule network in syncytia. New specific screens should identify new effectors targeting this key process for cell morphogenesis and pathogen response.

Finally, several RKN and CN effectors have been characterized that mimic and/or interfere with plant hormone peptide pathways (recently reviewed by Gheysen and Mitchum, 2019). Several CN effectors resemble the CLAVATA3 (CLV3)/ESR (CLE) hormones involved in controlling cell proliferation and differentiation. *In vitro* binding assays have confirmed the interaction of these CLE-like effectors with known receptors of plant CLE-peptides, such as CLAVATA2 (CLV2). The secretion of such hormone-mimicking peptides enables PPN to modulate root cell hormonal balance to promote feeding site formation. Additional effectors, such as the *H. schachtii* Hs19C07 and Hs10A07, have been shown to modulate auxin signaling, by interacting with the auxin transporter LAX3 (Lee et al., 2011), and by affecting the expression of auxin-responsive factors (ARFs) (Hewezi et al., 2015; see below), respectively, to facilitate feeding site formation.

## Host Cell Reprogramming Through the Modulation of Gene Expression

The morphological, structural and metabolic changes associated with the ontogenesis of nematode feeding cells require the extensive reprogramming of plant gene expression (Szakasits et al., 2009; Favery et al., 2016). Gene expression is regulated principally in the nucleus, and several effectors are thought to target the nuclei of the cells destined to become feeding cells, as they have predicted plant-like nuclear or nucleolar localisation signals, and some have been detected in the nucleus following ectopic expression in *Nicotiana benthamiana* leaves. Nuclear translocation in host cells has been demonstrated by immunolocalisation for only three RKN effectors: the *M. incognita* MiEFF1 (Jaouannet et al., 2012) and the *M. javanica* MjNULG1a (Lin et al., 2012) of unknown functions, and the *M. graminicola* MgGPP involved in plant defense suppression (Chen et al., 2017). All three were localized to giant cell nuclei. However, the targets of these effectors have yet to be characterized. Interestingly, some RKN and CN effectors have been shown to target key regulatory processes, including the epigenetic modification of histones, transcriptional regulation and mRNA splicing.

The Hs32E03 effector of *H. schachtii* alters the acetylation of histones by interacting with the *Arabidopsis* histone deacetylase (HDAC) HDT1 and FK506 binding protein, FKBP53 (Vijayapalani et al., 2018) in the nucleus. HDT1 and FKBP53 repress the transcription of rRNA genes, with HDT1 deacetylating histone H3 at Lys-9. Hs32E03 has been shown to inhibit HDAC, and an assessment of histone modifications in Hs32E03-expressing *Arabidopsis* lines based on chromatin immunoprecipitation revealed that these lines had abnormally high levels of acetylation in rDNA regions. As expected, rRNA levels were high in the line showing a low expression of Hs32E03 and displaying higher levels of CN infection. Interestingly, lower levels of rRNA were detected in the line highly expressing Hs32E03, due to the hypermethylation of rDNA promoters, resulting in an inhibition of nematode development. These findings highlight the importance of rRNA levels for syncytium formation, as protein overproduction is required, which in turn necessitates the synthesis of additional ribosomes. Hs32E03 is the first nematode effector for which a role has been reported in the epigenetic regulation of plant gene expression to promote parasitism.

Several other nuclear effectors have been shown to target transcription factors directly. The *M. incognita* Mi16D10 effector, which has a C-terminal CLE-like domain, interacts with SCARECROW-like transcription factors from both tomato and *Arabidopsis* (Huang et al., 2006). SCARECROW transcription factors are involved in root radial patterning, particularly in endoderm differentiation, and they act in concert with a short root transcription (SHR) factor (Hirsch and Oldroyd, 2009). Plants overexpressing Mi16D10 have larger root systems, implicating this effector in the modulation of root development. Another example is provided by the *H. schachtii* effector Hs10A07, which is secreted into the cytoplasm and then phosphorylated by an *Arabidopsis* kinase. This phosphorylation leads to its translocation into the nucleus, where it interacts with a second protein, IAA16, an Aux/IAA transcription factor, to modulate ARF expression (Hewezi et al., 2015).

Other effectors may modulate gene transcription directly by binding to DNA. Examples include *H. glycines* HgGLAND4 (Barnes et al., 2018) and the *M. incognita* 7H08 effector (Zhang et al., 2015). HgGLAND4 has been shown to bind specifically to the promoters of LTP genes implicated in plant defense, suppressing their expression (Barnes et al., 2018). Mi7H08 has been shown to be imported into the nucleus, and to activate the transcription of a reporter gene *in planta*, but the host genes regulated by this effector have yet to be identified (Zhang et al., 2015).

Finally, a *H. schachtii* effector, Hs30D08, has been shown to interfere with mRNA splicing, thereby altering gene expression in feeding sites (Verma et al., 2018). RNA splicing is required to remove introns from pre-mRNA and to join the protein-coding sequences (exons) together during the translation of mRNA into protein. Alternative splicing (AS) may occur, and this represents another way of regulating gene expression and increasing protein diversity. In *Arabidopsis*, 70% of genes may be alternatively spliced, and AS has been shown to play a significant role in plant development, and in responses to abiotic and biotic stresses (Reddy et al., 2013; Yang et al., 2014). Hs30D08 has been shown to interact with an actor of the spliceosome machinery, the auxiliary spliceosomal protein SMU2, in *Arabidopsis* (Verma et al., 2018). Transcriptomic analyses of *Arabidopsis* lines expressing the Hs30D08 confirmed its function in modulating AS and gene expression. Future investigations will shed light on the role of splicing and AS in feeding cell formation and plant responses to CN and RKN.

## Conclusion and Perspectives

The repertoire of putative RKN and CN effectors is extremely large, and proteinaceous effectors have been shown to target diverse compartments, manipulating many host plant functions to orchestrate the suppression of plant defenses, the formation of feeding sites and the promotion of nematode survival and reproduction. Moreover, the arsenal of plant pathogens is not restricted to proteinaceous effectors. They also secrete other molecules, such as secondary metabolites, glycolipids, hormones analogs, or small RNAs, to alter plant functions (Weiberg et al., 2013; Manosalva et al., 2015; Collemare et al., 2019). However, few data are available concerning the functions of effectors and the plant processes they target. The elucidation of effector function and the identification of host targets during parasitism thus remain major challenges. The large-scale identification of effector targets, particularly in crops, would be an important breakthrough potentially leading to the discovery of new processes involved in plant-nematode dialog. Comparison of RKN- and CN-targets will shed light on processes involved in their specific parasitic strategies and host ranges.

Functional analyses of effector targets may lead to the identification of susceptibility genes with potential for use in resistance breeding (De Almeida Engler et al., 2005; van Schie and Takken, 2014). In addition, “hubs,” susceptibility factors frequently targeted by different pathogens, may constitute ideal candidates for the design of broad-host range resistance in plants. However, these susceptibility genes are often crucial for plant physiology and development. Interfering with host protein recognition by pathogen effectors may be an interesting way of preserving important plant functions whilst breaking the susceptibility of the plant to pathogens. The breeding of new crops harboring point mutations that are less susceptible to diseases may be achieved with new technologies, such as the TILLING and CRISPR/Cas9 technologies, which are increasingly widely used (Engelhardt et al., 2018; Zaidi et al., 2018). Improvements in our understanding of effector/target functions are required if we are to block plant–microbe compatible interactions and engineer durable disease resistance.

## Author Contributions

All authors wrote the manuscript and approved it for publication.

## Conflict of Interest Statement

The authors declare that the research was conducted in the absence of any commercial or financial relationships that could be construed as a potential conflict of interest.
